# Physiotherapy Questionnaires App to Deliver Main Musculoskeletal Assessment Questionnaires: Development and Validation Study

**DOI:** 10.2196/rehab.9247

**Published:** 2018-02-23

**Authors:** Nestor Cavalcante Teixeira Neto, Yuri Lopes Lima, Gabriel Peixoto Leão Almeida, Márcio Almeida Bezerra, Pedro Olavo De Paula Lima, Rodrigo Ribeiro de Oliveira

**Affiliations:** ^1^ Department of Physical Therapy Faculty of Medicine Federal University of Ceará Fortaleza Brazil

**Keywords:** mobile phone, Foot and Ankle Outcome Score, American Orthopaedic Foot and Ankle Society, musculoskeletal assessment questionnaires, health survey

## Abstract

**Background:**

Patient-reported outcomes (PROs) translate subjective outcomes into objective data that can be quantified and analyzed. Nevertheless, the use of PROs in their traditional paper format is not practical for clinical practice due to limitations associated with the analysis and management of the data. To address the need for a viable way to group and utilize the main functioning assessment tools in the field of musculoskeletal disorders, the Physiotherapy Questionnaires app was developed.

**Objective:**

This study aims to explain the development of the app, to validate it using two questionnaires, and to analyze whether participants prefer to use the app or the paper version of the questionnaires.

**Methods:**

In the first stage, the app for an Android operational system was developed. In the second stage, the aim was to select questionnaires that were most often used in musculoskeletal clinical practice and research. The Foot and Ankle Outcome Score (FAOS) and American Orthopaedic Foot and Ankle Society (AOFAS) questionnaire were selected to validate the app. In total, 50 participants completed the paper and app versions of the AOFAS and 50 completed the FAOS. The study’s outcomes were the correlation of the data between the paper and app versions as well as the preference of the participants between the two versions.

**Results:**

The app was approved by experts after the adaptations of the layout for mobile phones and a total of 18 questionnaires were included in the app. Moreover, the app allows the generation of PDF and Excel files with the patients’ data. In regards to validity, the mean of the total scores of the FAOS were 91.54% (SD 8.86%) for the paper version and 91.74% (SD 9.20%) for the app. There was no statistically significant differences in the means of the total scores or the subscales (*P*=.11-.94). The mean total scores for the AOFAS were 93.94 (SD 8.47) for the paper version and 93.96 (SD 8.48) for the app. No statistically significant differences were found for the total scores for the AOFAS or the subscales (*P*>.99). The app showed excellent agreement with the paper version of the FAOS, with an ICC value of 0.98 for the total score (95% CI 0.98-0.99), which was also found for the AOFAS with the ICC for the total score of 0.99 (95% CI 0.98-0.99). For compliance, 72% (36/50) of the participants in the FAOS group and 94% (47/50) in the AOFAS group preferred the app version.

**Conclusions:**

The Physiotherapy Questionnaires app showed validity and high levels of compliance for the FAOS and AOFAS, which indicates it is not inferior to the paper version of these two questionnaires and confirms its viability and feasibility for use in clinical practice.

## Introduction

Patient-reported outcomes (PROs) [1] translate subjective outcomes, such as pain, function, daily activities, and social participation, into objective data that can be quantified and analyzed. Establishing quantitative parameters facilitates the diagnosis, prognosis, clinical decision making, and analysis of the progression of dysfunction and diseases [2]. Questionnaires are essential in research and clinical practice because they are efficient, reliable, and affordable [3].

Nevertheless, the use of PROs in their traditional paper format is not practical for clinical practice due to limitations associated with the analysis and management of the data [4,5]. Thus, in the last several decades, electronic patient-reported outcomes (ePROs) have been developed as an alternative [6]. Initially, ePROs were developed in Web platforms [7] and software programs that were accessed via personal computers [8]; however, mobile phones have added portability and viability to the tools used in health care. Currently, there is an increase in the use of mobile devices. It is estimated that one-third of the world’s population uses a mobile phone [9], which increases the use of new tools to measure people’s health status.

Today, approximately 40,000 mobile health (mHealth) apps are available; this suggests that there is a significant need for this kind of electronic assessment tool [10]. The advantages of the clinical use of a mobile phone app are the possibility of producing high-quality and reliable data using a little amount of space and the possibility of performing uploads and backups to prevent loss of data [11]. From a technical perspective, mobile phone apps offer large processing power, high-speed data transfer, and touchscreen resources, which avoid the use of paper, pens, and pencils. They are also printer-free, making their utilization more viable than paper [11]. Despite the wide availability of mHealth apps, an app with validated health care ePROS musculoskeletal data has not yet been developed.

To address the need for a viable way to group and utilize the main functioning assessment tools in the field of musculoskeletal disorders, the Physiotherapy Questionnaires app was developed. Paper versions of questionnaires are most often used; therefore, it is necessary to conduct a validation study to compare the paper and mobile phone versions of the physiotherapy questionnaires [12]. Thus, this study aims to explain the development of the app, to validate it using two physiotherapy questionnaires, and to analyze whether participants prefer to use the app or the paper version of the questionnaires.

## Methods

The study was conducted in two stages. In the first stage, the app for an Android operational system was developed. In the second stage, a pilot validation study of the app was conducted and user compliance was analyzed.

### Development of the App for Mobile Phones

The app is a collection of questionnaires related to clinical and functional diagnosis. As such, it aims to facilitate a feasible and portable assessment of musculoskeletal disorders. The app was coded by bachelor’s degree students in the Physical Therapy Program in partnership with students from the Computer Sciences program at the Federal University of Ceará, Fortaleza, Brazil, under the supervision of their professors. The Android platform was chosen for the app, using its native coding in Java in the Android Studio Integrated Development Environment with the Software Development Kit. This operational system was chosen due to the popularity and homogeneity of the hardware used on Android mobile phones.

In the initial stage of the development of the app, the aim was to select questionnaires that were most often used in musculoskeletal clinical practice and research. This selection was based on a literature search and input from a list of experts.

The trial version of the app was tested by 15 musculoskeletal physiotherapy fellows during their clinical practice to determine its feasibility. Weekly meetings were conducted over a period of 4 months so the experts could provide feedback about the possible difficulties in using electronic questionnaires. Based on their input, changes to the layout and functioning of the app were made.

### Validation of the App

The Foot and Ankle Outcome Score (FAOS) and the American Orthopaedic Foot and Ankle Society (AOFAS) questionnaire were randomly selected to validate the app. First, the ankle section was chosen, and then the questions in this section were randomized. After that, the FAOS and the AOFAS questionnaires were selected for inclusion in the app.

The FAOS was validated in Brazil. It aims to assess pain, symptoms, activities of daily living, and sports/recreation activities in subjects who have a sprained ankle despite the fact that this questionnaire is not specific for this condition [13]. The questionnaire is completely self-reported and it contains 42 questions.

The AOFAS also aims to assess the ankle region. This questionnaire is not considered to be a PRO because it is not completely self-reported; some questions require the intervention of the examiner. The AOFAS contains nine questions distributed into three categories: pain (40 points), function (50 points), and alignment (10 points), for a total of 100 points [14].

### Participants

A total of 100 participants were included in the study. The participants were males (n=30) and females (n=70) between the ages of 19 and 36 years (mean 24.2, SD 5.7 years). All participants signed an informed consent form. Participants who were unable to understand how to use the app were excluded from the study. The study was submitted to and approved by the Ethics Committee in Research with Human Beings of the Federal University of Ceará, Fortaleza, Brazil (number 1.847.143).

Both the paper and the electronic versions of the questionnaires were given to 50 participants for appropriate validation, thus resulting in a total of 100 participants [15]. The participants were divided in two groups: 50 completed the paper and app versions of the AOFAS and 50 completed the paper and app versions of the FAOS. The study’s outcomes were the correlation of the data between the paper and app versions as well as the preference of the participants between the two versions.

### Procedures

The data collection began after the participants signed the informed consent form. The paper and app versions were randomly distributed and the order of completion of each version was mixed. The time allotted to complete each version was determined. An interval of 15 minutes was established between the versions, as noted in the study by Ferrari et al [16]. After completing the questionnaires, the participants were asked: “Which method do you think was better to answer?” The participants had three possible ways to answer that question: (1) app, (2) paper, and (3) indifferent.

One researcher conducted a face-to-face assessment with all the participants using a previously structured explanation about how the app works and how to answer the paper version. In regards to the explanation on how to use the app, the participant was informed how to select the answer options and how to move to the following item in the questionnaire. After the participants completed the questionnaires on the mobile phone, the data were transferred to an email account that only the statistician had access to. After the data analysis was complete, all the information related to the questionnaires was removed from the device that was used to collect the data. Moreover, after the participants answered the paper version of the questionnaire, the examiner verified possible human errors and sent the data to the examiner responsible for the data extraction.

### Data Analysis

To check the validity of the two versions of the physiotherapy questionnaire (paper and electronic), the Wilcoxon *t* test was used to determine the differences between the means of the scores for the two versions and the intraclass correlation coefficient (ICC) was used to measure the level of intrarater reliability between the total scores, question by question in the AOFAS and by subscale in the FAOS, between the app and the paper versions. We considered ICC values ≥0.75 as excellent agreement and ICC values <0.75 as poor to moderate agreement [17]. Validity was defined by the correlation and the difference between the means of the scores of the two versions. The calculations were made using SPSS version 22.0 software for Windows, with a significance level of 5%.

## Results

### Development and Design of the App

A total of 18 clinical musculoskeletal-related questionnaires were included in the app. Thus, questionnaires relating to the ankle region (AOFAS, Foot And Ankle Ability Measure, FAOS, Lower Extremity Functional Scale, and Cumberland Ankle Instability Tool) [18-20], the knee (Victoria Institute Of Sport Assessment-Patella, Knee Instability Scale Modified For Evaluation Of Patellofemoral Pain And Instability, Fulkerson Scale, Lysholm Knee Scoring Scale, and Kujala Scoring Questionnaire or Anterior Knee Pain Scale) [21-24], the low back (Oswestry Low Back Pain Disability Questionnaire and Roland Morris Disability Questionnaire) [25], the shoulder (The Disabilities of the Arm, Shoulder and Hand Questionnaire, Shoulder Pain and Disability Index, Simple Shoulder Test, University of California at Los Angeles Shoulder Rating Scale, Western Ontario Rotator Cuff and American Shoulder and Elbow Surgeons Standardized Shoulder Assessment Form) [26,27], and the cervical spine (Neck Disability Index) [28] were included.

**Figure 1:**
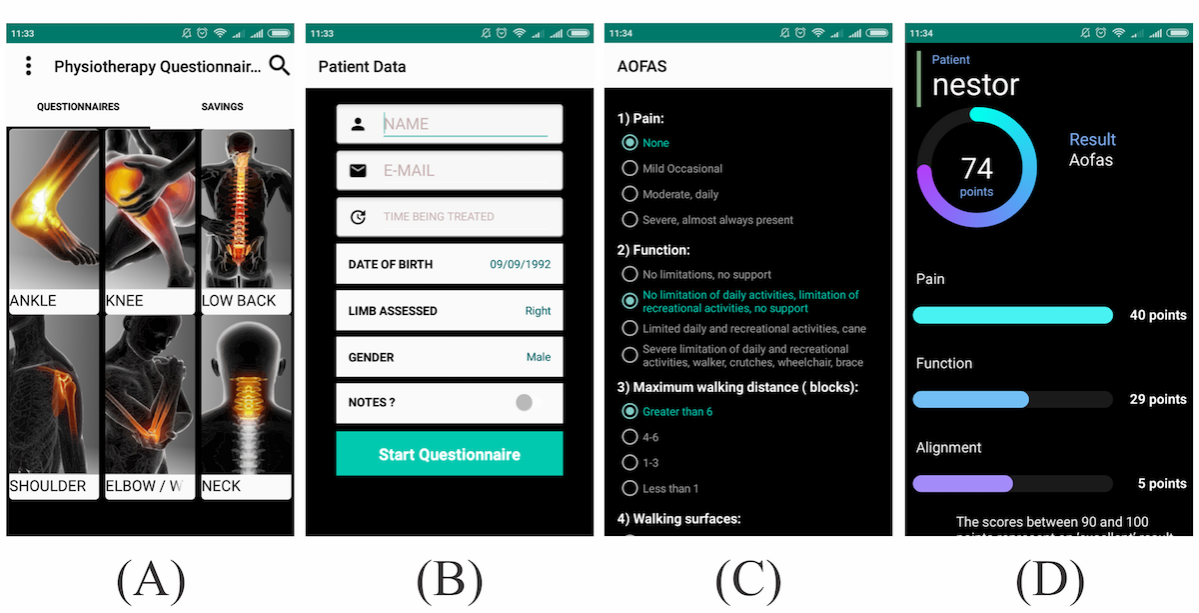
Screenshots of the Physiotherapy Questionnaires app. (A) Questionnaires by body region, (B) patient information record, (C) questionnaire, and (D) results.

**Figure 2:**
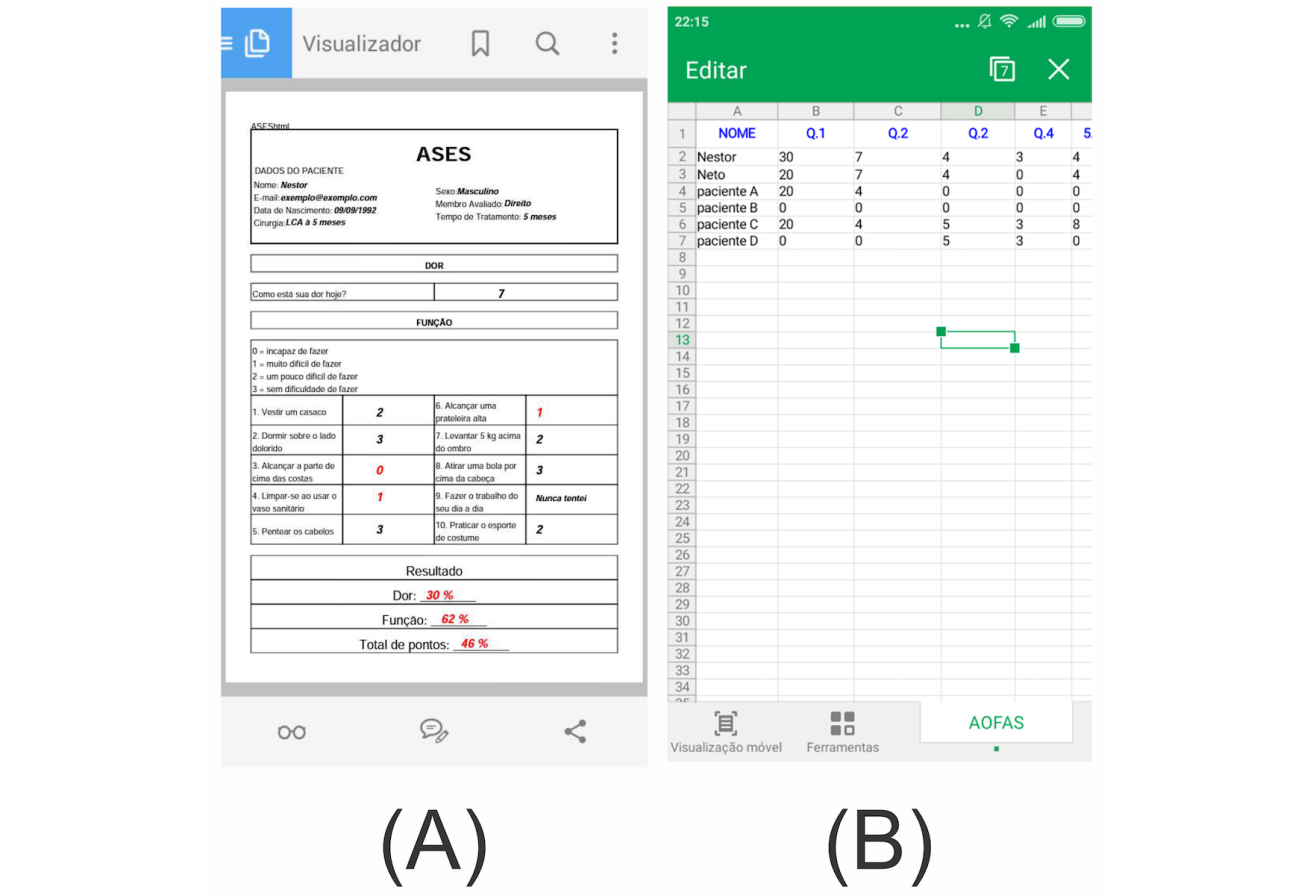
Example of (A) a PDF file and (B) an Excel file of a report containing all the user’s answers.

The final version of the app is available as a free download in English and Portuguese at the Google Play Store. In the first few days after release, more than 1000 apps were downloaded in Brazil. The app is basically composed of four screens. First, the user is presented with the categories of the questionnaires divided by body region (see Figure 1 A). The user must then click on one of the questionnaires to be redirected to a screen containing a patient information record (see Figure 1 B). Next, the questionnaire appears (see Figure 1 C). After the user has completed the questionnaire, the total score and the score by subscale are shown, informing the user about the scores and references for those scores (see Figure 1 D). Finally, on the results screen there is an option to generate a PDF file of a report containing all the user’s answers; the answers with a low score are highlighted (see Figure 2 A).

**Figure 3:**
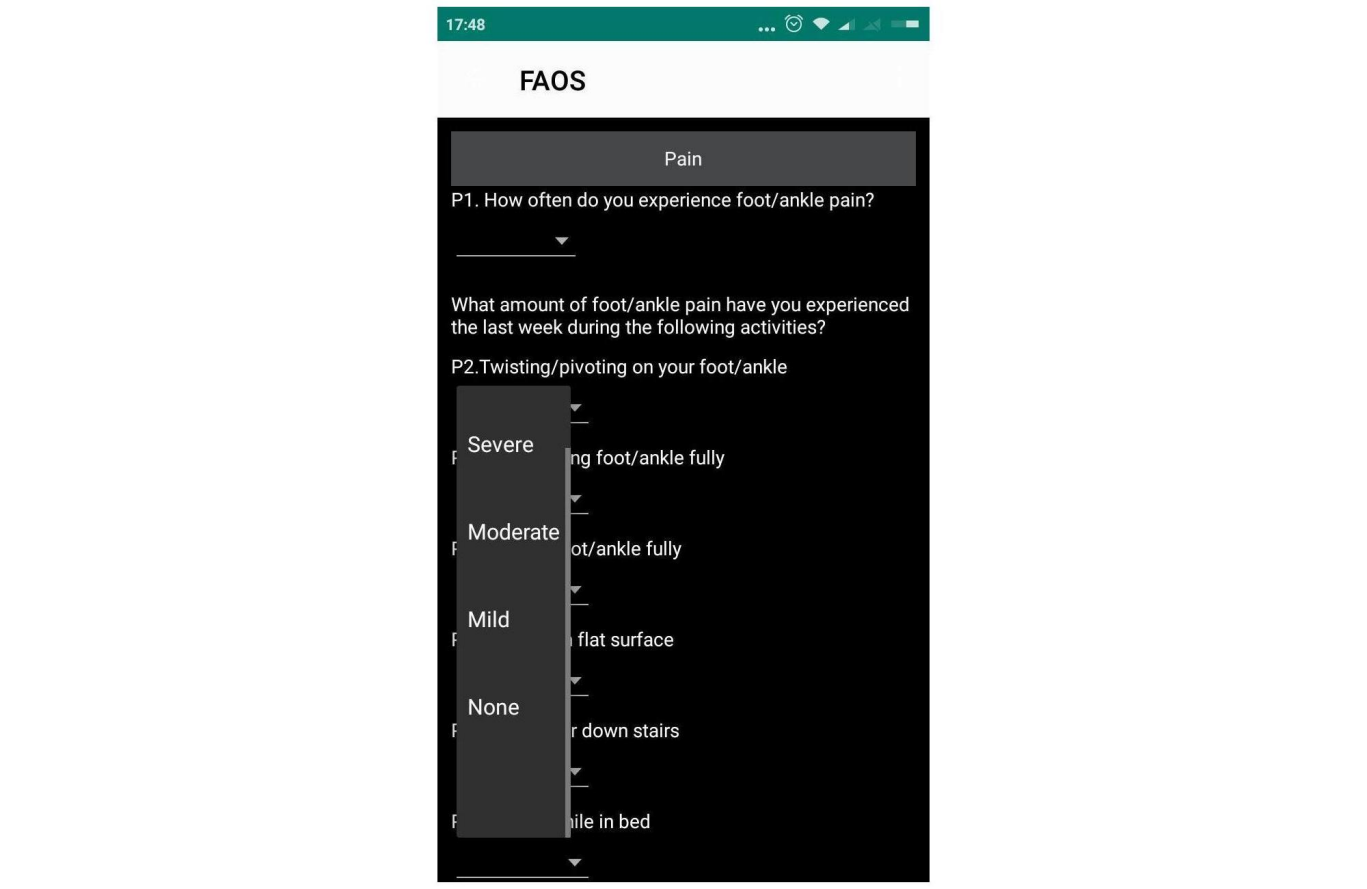
Screenshot of the app version of the Foot and Ankle Outcome Score (FAOS).

For the layout of the ePROs used for the pilot validation, the questions in the AOFAS were answered by touching one of four circles or, alternatively, touching the text (see Figure 1 B). The score was then assigned based on the validated paper version [14], generating a total score of 100 points. Conversely, in the FAOS, the alternatives were displayed in the form of spinners and they were ranked using a Likert scale (0 to 4), as shown in Figure 3. The total score and the score by subscale in the FAOS were converted to percentages, ranging from 0% to 100% [13].

To make it more feasible to use ePROs, the app was developed so that it would work offline to avoid usability issues due to poor internet connections. In addition to calculating the score, the electronic version of the physiotherapy questionnaires allowed users to generate an Excel file containing the values of all the items selected by the patients (see Figure 2 B), making the data collection and statistical analysis easier.

### Validating the App

The mean of the total scores of the FAOS were 91.54% (SD 8.86%) for the paper version and mean 91.74% (SD 9.20%) for the app, with a difference of 0.20% between the two versions. There was no statistically significant differences between the means of the total scores or the subscales (*P*=.11-.94). The means, the difference between the means, the standard deviations, and the *P* values for the scores for the paper and app versions for each question in the FAOS are presented in Table 1.

The app showed excellent agreement with the paper version of the FAOS, with an ICC value of 0.98 (95% CI 0.98-0.99) for the total score. The same level of agreement was found in the comparison between the paper version and the app version for each of the FAOS subscales, with the lowest ICC value of 0.93 (95% CI 0.88-0.96) in the Sports and Recreation subscale. The values of agreement for the FAOS subscales are listed in Table 1.

**Table 1:** Comparison between the paper and app versions of the Foot and Ankle Outcome Score (FAOS).

Header		Paper (%), mean (SD)		App (%), mean (SD)		Mean difference (%)		*P*		ICC (95% CI)	
Total		91.54 (8.86)		91.74 (9.12)		0.20		.23		0.98 (0.98-0.99)	
**Subscales**											
	Pain		91.34 (10.44)		90.96 (11.28)		0.38		.94		0.95 (0.91-0.97)
	Symptoms		88.70 (9.87)		89.22 (10.92)		0.52		.14		0.92 (0.86-0.95)
	Activities of daily living		95.98 (6.55)		96.22 (6.64)		0.24		.11		0.99 (0.98-0.99)
	Sports/recreation		85.10 (20.98)		86.90 (18.74)		1.80		.28		0.93 (0.88-0.96)
	Quality of life		86.54 (17.77)		86.78 (17.54)		0.24		.37		0.99 (0.99-0.99)

The mean total scores for the AOFAS were 93.94 (SD 8.47) for the paper version and mean 93.96 (SD 8.48) for the app, with a difference of 0.02 points between the two versions. The results were similar to the results for the FAOS; no statistically significant differences were found for the total scores for the AOFAS or the subscales (*P*>.99). The means, the difference between the means, the standard deviations, and the *P* values for the scores for the paper and app versions for each question in the AOFAS are presented in Table 2.

**Figure 4:**
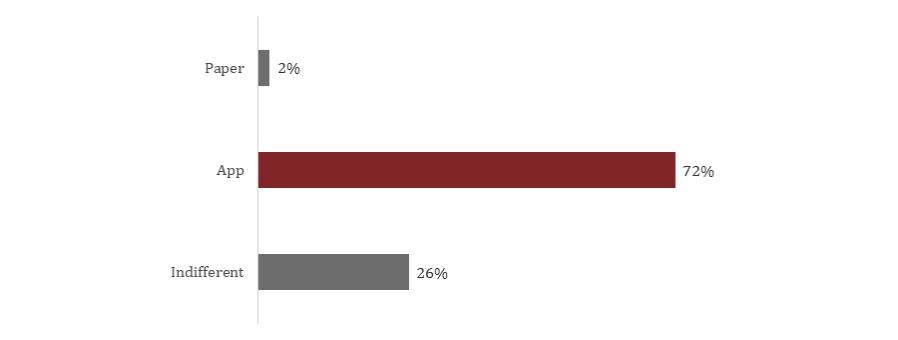
Preference of participants between the paper and app versions of the Foot and Ankle Outcome Score.

**Figure 5:**
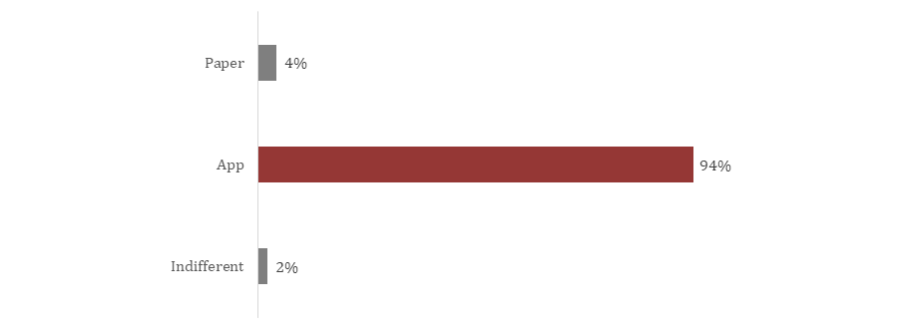
Preference of participants between the paper and app versions of the American Orthopaedic Foot and Ankle Society questionnaire.

Excellent agreement between the app version and the paper version was also found for the AOFAS. The ICC value for the total score in the AOFAS was 0.99 (95% CI 0.98-0.99). A similar level of agreement was also found for the AOFAS questions, with the lowest ICC value of 0.87 (95% CI 0.78-0.93) in question 3. It was not possible to calculate the correlation in question 6 because there was no variation in the score of this question between the paper and app versions. The values of agreement for the AOFAS questions are presented in Table 2. Details about the participants’ preferences are presented in Figures 4 and 5.

The mean time to complete the paper version of the FAOS was 170.18 (SD 47.30) seconds; the mean time to complete the app version was 189.50 (SD 69.61) seconds. Thus, the paper version was completed 19.32 seconds faster than the app version (*P*=.004). Conversely, the mean time to complete the paper version of the AOFAS was 83.82 (SD 42.27) seconds; the mean time to complete the app version of the AOFAS was 53.64 (SD 29.04) seconds. Thus, the app version was completed 30.18 seconds faster than the paper version (*P*<.001).

**Table 2:** Comparison between the paper and app versions of the American Orthopaedic Foot and Ankle Society (AOFAS) questionnaire.

Reliability		Paper (%), mean (SD)	App (%), mean (SD)	Mean difference (%)	*P*	ICC (95% CI)
Total		93.96 (8.47)	93.94 (8.48)	0.02	>.99	0.99 (0.98-0.99)
**Questions**						
	Q1	37.60 (4.76)	37.60 (4.76)	0.00	>.99	1.00 (1.00-1.00)
	Q2	9.82 (0.59)	9.98 (0.59)	0.06	>.99	0.88 (0.79-0.93)
	Q3	4.88 (0.32)	4.84 (0.50)	0.04	>.99	0.87 (0.78-0.93)
	Q4	4.44 (0.90)	4.44 (0.90)	0.00	>.99	1.00 (1.00-1.00)
	Q5	7.78 (0.88)	7.78 (0.88)	0.00	>.99	1.00 (1.00-1.00)
	Q6	8.00 (0.00)	8.00 (0.00)	0.00	>.99	—^a^
	Q7	5.94 (0.42)	5.94 (0.42)	0.00	>.99	1.00 (1.00-1.00)
	Q8	7.20 (2.42)	7.20 (2.42)	0.00	>.99	1.00 (1.00-1.00)
	Q9	8.36 (2.51)	8.46 (2.55)	0.10	>.99	0.98 (0.96-0.98)

^a^The variables have zero variance.

## Discussion

### Principal Results

There is a lack of validated ePROs to help clinicians apply musculoskeletal measurement tools, especially in Portuguese [29]. This study aimed to develop an app and conduct a validation of two questionnaires used in the electronic version of the app. Although the wording of the questions in the proposed app was preserved, their layouts were adapted for use on mobile phones and the new format was approved by a list of experts as well as less-experienced patients. In this way, it is possible to ensure that the development of the app was successful.

The results of the study provide evidence about the agreement between the scales included in the paper and electronic versions in the PQapp. Although in a previous study Bierbrier et al [30] did not focus on musculoskeletal measurement tools, they did demonstrate the accuracy of the results of the app version of the physiotherapy questionnaire scales for mobile phones by testing a variety of mHealth apps obtained from iTunes and the Google Play Store. These data are important to ensure the reliability of the information obtained within the app, thus eliminating the possibility of human error. The results also provide important information about the validity of the reports generated by the electronic version of the AOFAS and the FAOS questionnaires [18] that are used to measure ankle instability.

The analysis of equivalence between an ePRO and its paper version can be conducted based on the degree of modification that was made to the electronic version [17]. In this study, it was necessary to moderately adapt the ePROs to create the app; this included reducing the font size and adding the use of ScrollView (scroll down to see all the alternatives) because some of the questions required more than one page. These modifications justify the need to conduct a validation study for the app. Due to logistical requirements and time unavailability for the validation of all questionnaires, our study is only a partial validation of the app. Thus, we decided to start by randomly choosing two questionnaires for the app: FAOS and AOFAS.

The concurrent validity of the FAOS and AOFAS questionnaires was supported by a strong positive correlation between the reports provided by the two different versions. The paper and mobile phone reports of other questionnaires have already been compared, and a high correlation between them has been found without any statistically significant differences. Bush et al [31] assessed active military personnel in the United States and reported similar answers for the means of seven dimensions between an app and a paper version of the questionnaire. Similarly, Garcia-Palacios et al [32] investigated the use of questionnaires for patients with fibromyalgia and reported no statistically significant differences between the means of the pain and fatigue scores obtained with the mobile phone and paper versions of the tool used in the study. For patients with rheumatic diseases, ePROs have been found to have excellent agreement with their paper versions [33]. Kim et al [34] found a strong correlation between paper and mobile phone versions of the International Prostate Symptom Score in their study of validity and reliability.

Only a few studies have compared the mobile phone and paper versions of questionnaires related to musculoskeletal conditions [29]. The initial validation process for the app demonstrates that FAOS and AOFAS are ePRO pioneers for ankle and foot assessments on mobile phones. In their electronic versions, both questionnaires present equivalent data, as recommended by Belisário et al [29]

In regard to the use of touchscreen technology in questionnaires that assess health outcomes, the evidence suggests that patients prefer electronic methods rather than paper because the information can be provided more efficiently and accurately than the paper version; the electronic version also guarantees increased safety when answering questionnaires on a mobile phone [29,33]. It has also been reported that it is safer and faster to answer the questionnaires on a mobile phone [34,35]. The results of our study confirm that users prefer to answer questionnaires using the app version instead of the paper version of the questionnaires.

**Figure 6:**
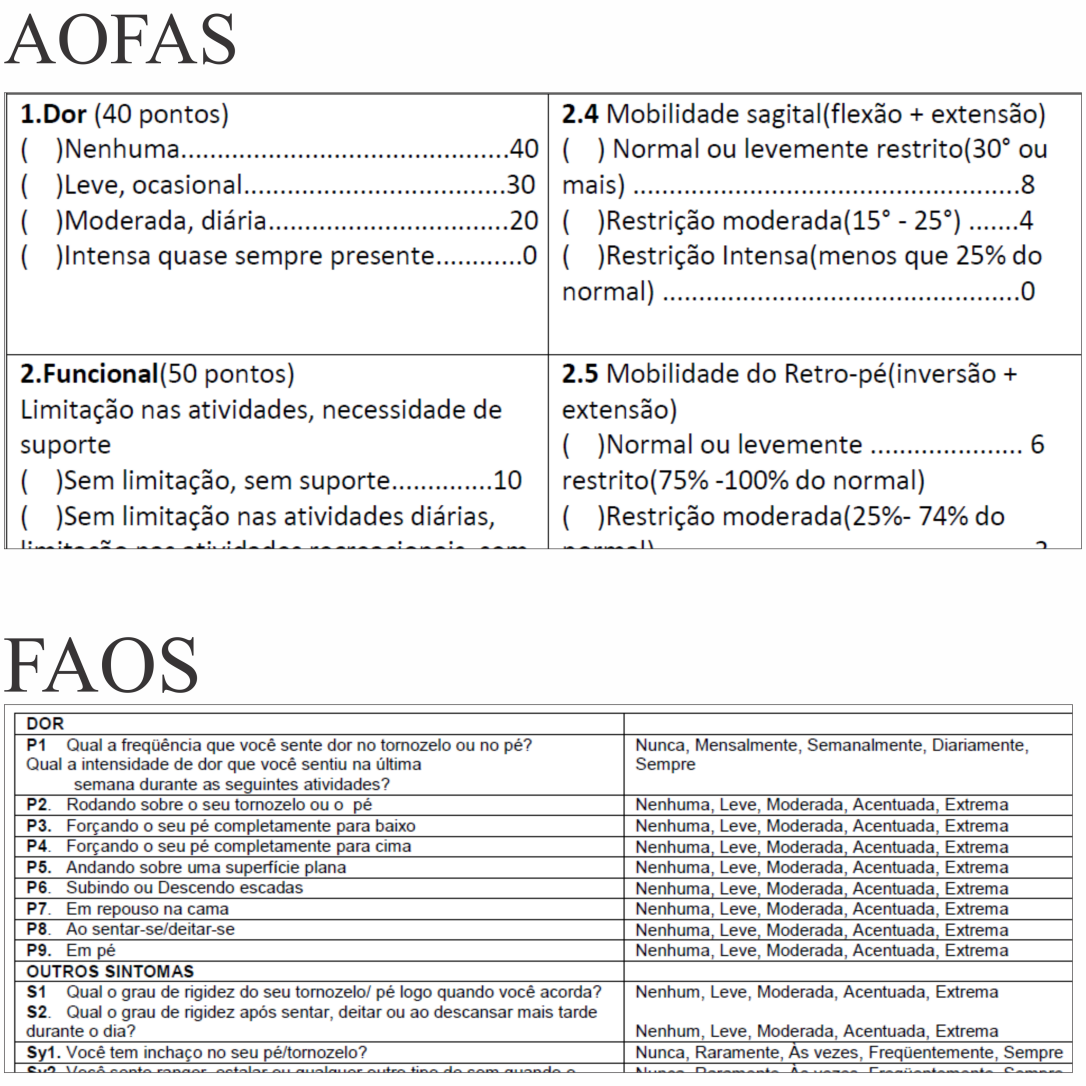
Paper versions (in Portuguese) of the American Orthopaedic Foot and Ankle Society (AOFAS) questionnaire and the Foot and Ankle Outcome Score (FAOS).

Belisário et al [29] affirmed that it is unclear whether it takes less time to complete a questionnaire on mobile phones than when using the paper version; however, they concluded that factors such as population characteristics, study design, and platform interface could have some effect on the result. In fact, our research showed a faster completion time for the paper version compared to the app version for the FAOS. This fact may be due to differences in the layout between the paper and app versions of this questionnaire. The alternatives were displayed in the form of spinners in the app (see Figure 3), which were revealed after the user clicked on the screen. In the paper version (see Figure 6), the alternatives were placed next to each other, which may have contributed to the faster completion time. Nevertheless, despite the statically significant difference of 19.32 seconds between the two versions, this value may not be clinically relevant.

### Limitations

This study has some limitations. Regarding the analysis of participant preferences, the graphic content and layout aspects of the app were not evaluated during the data collection. Thus, future research might consider these factors to obtain a better assessment of participant satisfaction.

Moreover, we did not evaluate the experience/familiarity of the participants with mobile phones. Nevertheless, we believe that the high ICC values might be due to the fact that only young participants who knew how to operate a mobile phone were included in the study. This might have resulted in selection bias; however, this is the first study to show the validity of a mobile phone version of the FAOS and AOFAS questionnaires using scientific methods.

### Conclusions

The Physiotherapy Questionnaires app is a useful tool for health care professionals because it combines two main questionnaires used to assess musculoskeletal disorders. The app allows clinicians to easily and effectively calculate, save, and organize the patient’s answers to two physiotherapy questionnaires. The app showed validity and high levels of compliance for the FAOS and AOFAS, which indicates it is not inferior to the paper version of these two questionnaires and confirms the app’s viability and feasibility for use in clinical practice.

## Appendix

Multimedia Appendix 1 FAOS Questionnaire.

Multimedia Appendix 2 AOFAS Questionnaire.

Multimedia Appendix 3 Video promo pq app.

## Abbreviations

AOFAS: American Orthopaedic Foot and Ankle Society
ePRO: electronic patient-reported outcome
FAOS: The Foot and Ankle Outcome Score
ICC: interclass correlation coefficient
mHealth: mobile health
PRO: patient-reported outcome
