# Supplementary material for: Physiotherapy Questionnaires App to Deliver Main Musculoskeletal Assessment Questionnaires: Development and Validation Study
Source: JMIR Rehabil Assist Technol. 2018 Feb 23;5(1):e1. doi: 10.2196/rehab.9247 (PMC5845103; doi:10.2196/rehab.9247)
Supplement: Multimedia Appendix 1 [file rehab_v5i1e1_app1.pdf]

FAOS – Questionário para pé e tornozelo

Paciente: ____ ______________ **Aplicado por: (nº crachá)**_________
Diagnóstico: ____**Data:** _____________

Tradução do FAOS 42 itens estão subdivididos em 5 subescalas: Dor, Outros Sintomas, Atividades de Vida Diária, Atividades esportivas e recreacionais e Qualidade de Vida relacionada ao pé e tornozelo. As 5 opções de resposta são dadas após cada item.

**DOR**		**Pontuação**
**P1** Qual a freqüência que você sente dor no tornozelo ou no pé?<br>Qual a intensidade de dor que você sentiu na última semana durante as seguintes atividades?	Nunca, Mensalmente, Semanalmente, Diariamente, Sempre	
**P2**. Rodando sobre o seu tornozelo ou o pé	Nenhuma, Leve, Moderada, Acentuada, Extrema	
**P3.** Forçando o seu pé completamente para baixo	Nenhuma, Leve, Moderada, Acentuada, Extrema	
**P4**. Forçando o seu pé completamente para cima	Nenhuma, Leve, Moderada, Acentuada, Extrema	
**P5.** Andando sobre uma superfície plana	Nenhuma, Leve, Moderada, Acentuada, Extrema	
**P6**. Subindo ou Descendo escadas	Nenhuma, Leve, Moderada, Acentuada, Extrema	
**P7**. Em repouso na cama	Nenhuma, Leve, Moderada, Acentuada, Extrema	
**P8**. Ao sentar-se/deitar-se	Nenhuma, Leve, Moderada, Acentuada, Extrema	
**P9.** Em pé	Nenhuma, Leve, Moderada, Acentuada, Extrema	
**OUTROS SINTOMAS**		
**S1** Qual o grau de rigidez do seu tornozelo/ pé logo quando você acorda?<br>**S2**. Qual o grau de rigidez após sentar, deitar ou ao descansar mais tarde durante o dia?	Nenhum, Leve, Moderada, Acentuada, Extrema<br>Nenhum, Leve, Moderada, Acentuada, Extrema	
**Sy1.** Você tem inchaço no seu pé/tornozelo?	Nunca, Raramente, Às vezes, Freqüentemente, Sempre	
**Sy2**. Você sente ranger, estalar ou qualquer outro tipo de som quando o movimenta o pé?	Nunca, Raramente, Às vezes, Freqüentemente, Sempre	
**Sy3.** O seu pé trava ou fica bloqueado aos movimentos?	Nunca, Raramente, Às vezes, Freqüentemente, Sempre	
**Sy4.** Você consegue forçar o seu pé completamente para baixo?	Sempre, Freqüentemente, Às vezes, Raramente, Nunca	
**Sy5**. Você consegue forçar o seu pé completamente para cima?	Sempre, Freqüentemente, Às vezes, Raramente, Nunca	
**ATIVIDADES DE VIDA DIÁRIA**<br>Qual a dificuldade que você sentiu nesta última semana :		
**A1.** Descendo escadas	Nenhuma, Leve, Moderada, Acentuada, Extrema	
**A2**. Subindo escadas	Nenhuma, Leve, Moderada, Acentuada, Extrema	
**A3**. Levantando-se a partir da posição sentada	Nenhuma, Leve, Moderada, Acentuada, Extrema	
**A4**. Em pé	Nenhuma, Leve, Moderada, Acentuada, Extrema	
**A5**. Curvando-se para pegar um objeto no chão	Nenhuma, Leve, Moderada, Acentuada, Extrema	
**A6**. Andando em superfícies planas	Nenhuma, Leve, Moderada, Acentuada, Extrema	
**A7**. Entrando e saindo do carro	Nenhuma, Leve, Moderada, Acentuada, Extrema	
**A8**. Indo às compras	Nenhuma, Leve, Moderada, Acentuada, Extrema	
**A9.** Colocando meias	Nenhuma, Leve, Moderada, Acentuada, Extrema	
**A10**. Levantando-se da cama	Nenhuma, Leve, Moderada, Acentuada, Extrema	
**A11** Tirando as meias	Nenhuma, Leve, Moderada, Acentuada, Extrema	
**A12 V**irando-se na cama mantendo a mesma posição do tornozelo/pé	Nenhuma, Leve, Moderada, Acentuada, Extrema	
**A13**. Entrando ou saindo do banho	Nenhuma, Leve, Moderada, Acentuada, Extrema	
**A14** Sentando	Nenhuma, Leve, Moderada, Acentuada, Extrema	
**A15**. Sentando e levantando do vaso sanitário	Nenhuma, Leve, Moderada, Acentuada, Extrema	
**A16.** Realizando tarefas domésticas pesadas (deslocando caixas pesadas, esfregando o chão, etc)	Nenhuma, Leve, Moderada, Acentuada, Extrema	
**A17.** Realizando tarefas domésticas leves (cozinhando, varrendo, barbeando-se etc)	Nenhuma, Leve, Moderada, Acentuada, Extrema	
**Atividades ESPORTIVAS E RECREACIONAIS**<br>Qual a dificuldade que você sentiu ou sentiria nesta última semana :		
**Sp1**. Agachando	Nenhuma, Leve, Moderada, Acentuada, Extrema	
**Sp2**. Correndo	Nenhuma, Leve, Moderada, Acentuada, Extrema	
**Sp3**. Pulando	Nenhuma, Leve, Moderada, Acentuada, Extrema	
**Sp4**. Mudando de direção sobre o seu tornozelo/.pé lesionado	Nenhuma, Leve, Moderada, Acentuada, Extrema	
**Sp5.** Ajoelhando-se	Nenhuma, Leve, Moderada, Acentuada, Extrema	
**QUALIDADE DE VIDA EM RELAÇÃO AO PÉ E TORNOZELO**<br>**Q1.** Com que freqüência que você percebido os problemas do seu tornozelo/pé?	Nunca, Mensalmente, Semanalmente, Diariamente, Sempre	
**Q2**. Você tem modificado seu estilo de vida para evitar atividades potencialmente danosas para o seu tornozelo/pé?	Não, um pouco, Moderamente, Muito, totalmente	
**Q3**. O quanto você está incomodado com a falta de confiança no seu pé/tornozelo?	Não, Um pouco, Moderamente, Muito, Totalmente	
**Q4**. No geral, qual grau de dificuldade você tem com o seu tornozelo/pé?	Nenhum, Leve, Moderada, Acentuada, Extrema	
