# Supplementary material for: Physiotherapy Questionnaires App to Deliver Main Musculoskeletal Assessment Questionnaires: Development and Validation Study
Source: JMIR Rehabil Assist Technol. 2018 Feb 23;5(1):e1. doi: 10.2196/rehab.9247 (PMC5845103; doi:10.2196/rehab.9247)
Supplement: Multimedia Appendix 2 [file rehab_v5i1e1_app2.pdf]

Nome: ________________________________

Celular: ________________ E-mail:______________________

Qual versão foi respondida primeira? App ( ) ou Papel ( )

Qual versão você gostou mais? App ( ) ou Papel ( )

AOFAS	
**1.Dor** (40 pontos)<br>( )Nenhuma...40<br>( )Leve, ocasional..................................30<br>( )Moderada, diária...............................20<br>( )Intensa quase sempre presente...........0	**2.4** Mobilidade sagital(flexão + extensão)<br>( ) Normal ou levemente restrito(30° ou mais) ....8<br>( )Restrição moderada(15° - 25°) ....4<br>( )Restrição Intensa(menos que 25% do normal) 0
**2.Funcional**(50 pontos)<br>Limitação nas atividades, necessidade de suporte<br>( )Sem limitação, sem suporte.............10<br>( )Sem limitação nas atividades diárias, limitação nas atividades recreacionais, sem suporte..7<br>( )Limitação nas atividades diárias e recreacionais, bengala.............................4<br>( )Limitação intensa nas atividades diárias e recreacionais, andador, muletas, cadeira-de-rodas, órtese(tornozeleira, imobilizador de tornozelo) 0	**2.5** Mobilidade do Retro-pé(inversão + extensão)<br>( )Normal ou levemente .... 6 restrito(75% -100% do normal)<br>( )Restrição moderada(25%- 74% do normal) .... 3<br>( ) Restrição intensa(menos que 25% do normal) 0
**2.1** Distância máxima de caminhada, quarterões<br>( ) Mais que 6.......................................5<br>( ) De 4 a 6...4<br>( ) De 1 a 3...2<br>( ) Menos que 1....................................0	**2.6** Estabilidade do tornozelo e retro-pé(anteroposterior, varo-valgo)<br>( ) Estável .... 8<br>( ) Instável 0
**2.2** Superfícies de caminhada<br>( ) Sem dificuldades em qualquer superfície..5<br>( )Algumas dificuldades em terrenos irregulares, escadas, inclinações e ladeiras..3<br>( ) Dificuldade intensa em terrenos irregulares, escadas, inclinações e ladeiras..0	**3. Alinhamento** (10 pontos)<br>( ) Bom, pé plantígrado, ante-pé e retro-pé bem alinhado .... 10<br>( ) Regular, pé plantígrado, algum grau de desalinhamento do tornozelo e retro-pé, sem sintomas .... 5<br>( ) Ruim, pé não plantígrado, desalinhamento intenso e sintomático .. 0
**2.3** Anormalidade na macha<br>( ) Nenhuma, leve.................................8<br>( ) Evidente..4<br>( ) Acentuada..0	**Total de pontos: __________**
